# A comparative study of modified confirmatory techniques and additional immuno-based methods for non-conclusive autolytic Bovine spongiform encephalopathy cases

**DOI:** 10.1186/1746-6148-9-212

**Published:** 2013-10-18

**Authors:** Rocío Sarasa, Dietmar Becher, Juan J Badiola, Marta Monzón

**Affiliations:** 1 Research Centre for Encephalopathies and Transmissible Emerging Diseases, University of Zaragoza, Zaragoza, Spain; 2 Micromun Privates Institut für Mikrobiologische Forschung GmbH, Greifswald, Germany

**Keywords:** TSEs, BSE, Confirmatory diagnosis, Non-conclusive cases

## Abstract

**Background:**

In the framework of the Bovine Spongiform Encephalopathy (BSE) surveillance programme, samples with non-conclusive results using the OIE confirmatory techniques have been repeatedly found. It is therefore necessary to question the adequacy of the previously established consequences of this non-conclusive result: the danger of failing to detect potentially infected cattle or erroneous information that may affect the decision of culling or not of an entire bovine cohort. Moreover, there is a very real risk that the underreporting of cases may possibly lead to distortion of the BSE epidemiological information for a given country.

In this study, samples from bovine nervous tissue presenting non-conclusive results by conventional OIE techniques (Western blot and immunohistochemistry) were analyzed. Their common characteristic was a very advanced degree of autolysis. All techniques recommended by the OIE for BSE diagnosis were applied on all these samples in order to provide a comparative study. Specifically, immunohistochemistry, Western blotting, SAF detection by electron microscopy and mouse bioassay were compared. Besides, other non confirmatory techniques, confocal scanning microscopy and colloidal gold labelling of fibrils, were applied on these samples for confirming and improving the results.

**Results:**

Immunocytochemistry showed immunostaining in agreement with the positive results finally provided by the other confirmatory techniques. These results corroborated the suitability of this technique which was previously developed to examine autolysed (liquified) brain samples. Transmission after inoculation of a transgenic murine model TgbovXV was successful in all inocula but not in all mice, perhaps due to the very scarce PrPsc concentration present in samples.

Electron microscopy, currently fallen into disuse, was demonstrated to be, not only capable to provide a final diagnosis despite the autolytic state of samples, but also to be a sensitive diagnostic alternative for resolving cases with low concentrations of PrPsc.

**Conclusions:**

Demonstration of transmission of the disease even with low concentrations of PrPsc should reinforce that vigilance is required in interpreting results so that subtle changes do not go unnoticed. To maintain a continued supervision of the techniques which are applied in the routine diagnosis would prove essential for the ultimate eradication of the disease.

## Background

Bovine Spongiform Encephalopathy (BSE) belongs to a group of neurodegenerative diseases termed Transmissible Spongiform Encephalopathies (TSEs). It is characterized by a chronic course (4–5 years of incubation period) and lethal end [1]. The causal agent is accepted to be an abnormally folded prion protein [2], which derives from a cellular protein (PrPc) which is present in the nervous tissue under physiological conditions and which, owing to a conformational change, turns into a pathological protein (PrPsc) presenting new features as insolubility [1], resistance to digestion [3] and exposure of some different epitopes [4].

Accumulation of PrPsc and bilaterally symmetrical vacuolization of the neuropil and/or neuronal perikaryon [5] are specific TSEs features. Deposits of PrPsc appear exclusively in affected brains, being therefore considered as a pathognomonic finding [6] and are usually used as tool for diagnostic aims. PrPsc detection is possible due to its partial resistance to proteinase K digestion and shows a characteristic glycoform pattern by applying Western blotting techniques. PrPsc deposits appear along with neuronal loss and gliosis, even prior to the clinical symptoms [7] which mainly consist of apprehension or aggressive behavior, ataxia, trouble standing up, decreased milk production and weight loss [8].

Although BSE typically affects cattle, a rather low transmission barrier allows its transmission through inoculation or ingestion of affected tissues [9] to mice [5], which are used for diagnostic aims (bioassay). It can also affect human beings causing a variant of Creutzfeldt-Jakob disease (vCJD) [10]. Because of this fact, since the detection of the first case of BSE in 1986 [11] and subsequent increase of the number of affected cows and humans, apparently due to the contamination of cattle fed with PrPsc [12], an intensive surveillance programme was established to prevent the entry of contaminated material to the alimentary chain. All bovine fallen stock and slaughtered animals of a determined age have to be analyzed by rapid techniques [13] in order to provide a first rapid diagnosis. As these techniques can present a lower sensitivity than confirmatory techniques, subsequently, all samples giving a positive or non-conclusive result by rapid tests needed to be finally diagnosed by any of the confirmatory techniques as recommended by the *Office International des Epizooties*[14]: histological assessment, immunohistochemistry, Western blotting, Scrapie-associated fibrils (SAF) visualization by electron microscopy or bioassay for transmissibility of the disease.

However, problems arise in relation to samples which cannot be diagnosed by those confirmatory techniques applied for BSE diagnosis when there is a positive or non-conclusive result in routine tests. All and each one of these techniques were assessed in this work by using several samples which initially showed non-conclusive results, allowing a comparative analysis which has not been carried out previously.

Throughout this intensive programme, contrary to the initial idea of only one BSE strain [15], new strains could be identified presenting different behaviors with respect to classical BSE [16,17].

The objective proposed in this study is not only to confirm the positive or negative diagnosis of these samples, but also to find out whether the cause of their non-conclusive behavior by conventional techniques, could be related to a new strain of the causal agent which might be interfering in the result of these techniques.

## Methods

The samples included in this study were 5 bovine nervous tissues corresponding to the Spanish active surveillance programme which had presented a non-conclusive result by immunohistochemistry and Western blotting routinely applied for confirming BSE cases. All the samples were characterized by a very advanced degree of autolysis (liquified brains).

All the confirmatory techniques established by the OIE (immunohistochemistry, Western blotting, SAF visualization by electron microscopy and bioassay in a murine model) were applied on problem non-conclusive samples and were modified in order to increase the sensitivity to detect foreseeable low concentrations of PrPsc.

All these techniques were first set up on healthy and BSE non autolytic samples prior to be applied on autolytic control healthy and the five non-conclusive samples afterwards.

### Immunocytochemistry

The protocol applied was based on that described by Monleón et al. [18] for samples presenting a very advanced degree of autolysis (liquid state). The antibodies used were L42 (1/500, R-Biopharm, Darmstadt, Germany), R145 (1/500, DEFRA, UK) and F89 (1/2000, VMRD Inc, USA). Briefly, after dilution of sample in TBS, a 40 μl subaliquot from this dilution together with two subsequent serial decimal dilutions were dispensed into Vectabond-pretreated glass slides. Twenty four hour drying at 56°C and immersion into formalin 10% for 1 hour were applied prior to pre-treatment with 98% formic acid, proteinase K digestion and hydrated autoclaving before PrPsc detection using the monoclonal antibody stated above (RT for 30 minutes). EnVision™ (DAKO, Denmark) was used as visualization system and DAB as chromogen.

In order to enhance the immunosignal for PrPsc, a modification of the protocol consisting of the addition of a commercial linker (DAKO, Denmark) during 15 min before the addition of the primary antibody and a specific visualization system (EnVision™ FLEX/HRP, DAKO, Denmark) during 30 min before DAB visualization (DAKO, Denmark) was also assessed.

### Western blotting

The protocol followed was that published by the OIE [19] by using 6H4 as primary antibody (1:2500; Prionics, Zurich, Switzerland) since it showed the most sensitivity.

In order to increase the sensitivity of the technique and to assure that PrPsc concentration was enough for its detection, a purification and concentration treatment, combining the protocols described by Hilmert and Diringer [20], Hope et al. [21] and Stack et al. [22], was included. This finally consisted of a homogenization in 10% sarcosyl in water (pH 7.4 adjusted with NaH_2_PO_4_) and an incubation (RT for 30 min) before adding N-octanol to remove the foam. The homogenate was centrifuged (22.000 g for 30 min) and the pellet was subsequently diluted in 1% sarcosyl and 10% NaCl. This step was repeated with another centrifugation (215.000 g for 30 min) and a subsequent dilution of the pellet in 1 ml of 1% sarcosyl and 10% NaCl (1 h, 37°C) was carried out. After centrifugation (9.100 g for 15 min), the pellet diluted in the same solution was digested by proteinase K (Roche, Switzerland; 5 μg, 2 h at 37°C). A last centrifugation (9.100 g for 15 minutes) was required to obtain the pellet which was finally resuspended in water and subjected to the protocol of electrophoresis and immunotransfer detailed by the OIE.

Differentiation Western blotting technique (Bio Rad, Ref 3551177), following the manufacturer’s protocol, was also applied on all bovine samples with the aim of providing their glycoform patterns and trying to identify the causal strain in all cases.

### SAF visualization by electron microscopy

Prior to the application of the protocol based on that described by Narang and Perry [23], the same protocol of purification and concentration used for Western blotting methodology was used for SAF visualization.

For immunohistochemistry with colloidal gold, the protocol described by Merz et al. [24] was applied but using L42 as the primary antibody (1/500; R-Biopharm, Darmstadt, Germany) after following similar purification and concentration protocol. The size of the colloidal gold used was 10 nm.

### Bioassay in murine model

Transgenic mice TgbovXV [25] were intracranially and intraperitoneally inoculated with 20 μl and 100 μl from each sample, respectively. The inocula had to be diluted with physiological saline to a final concentration between 1.5% and 10% and needed to be heat treated (70°C for 15 min) to eliminate the very high cytotoxicity (in *in vitro* and *in vivo* assays) and microbial contamination present in all the samples. The average doses of brain material finally inoculated per mouse was between 1.7 mg and 7.2 mg.

During the incubation period, the animals were clinically examined once every day during the first month and twice a week until the end of the experiment. All the surviving animals were euthanized 730 days post-inoculation (DPI), except for those cases when the endpoint criteria dictated a premature euthanasia.

Rules established by the German Research Council’s guide for animal experimentation, based on European guidelines, were followed with respect to animal handling and care (AKTENZEINCHEN: LALLF M-V/TSD/7221.3-2.5-006/07).

Collected brains were divided into two portions, one fresh part for rapid techniques (BetaPrion BSE EIA Test Kit, Roboscreen, Germany and PLATELIA BSE Detection Kit, Bio-Rad, USA) and the other half was fixed in formalin (10%), which was transversally sliced in 5 sections [26], for confirmatory techniques.

#### Immunohistochemistry on murine samples

A protocol based on that described by Hortells et al. [27] was applied on each one of the 5 sections from all murine samples. Briefly, after pre-treatment (98% formic acid for 15 min, proteinase K digestion for 15 min at 37°C and hydrated autoclaving for 2 min) and endogenous peroxidase inhibition (0,03%, 8 min), the samples were incubated (30 min at RT) with primary antibody: 6H4 (1/100, Prionics, Zurich, Switzerland), R145 (1/100, DEFRA, UK) or F89 (0.5 μg/ml VMRD Inc, USA). Envision + TM Peroxidase (DAKO; Denmark) as the visualization system and DAB (5 min, 30%; DAKO, Denmark) as chromogen were used.

A variation of this immunohistochemical protocol consisting of linker addition (DAKO, Denmark) was also incorporated as described for bovine samples in order to assess whether the sensitivity could increase by using this variation.

Moreover, an ultrasensitive protocol for immunohistochemistry, Catalyzed Signal Amplification (CSA) System (DAKO, Denmark), was further assessed according to the manufacturers’ instructions with the same aim of trying to increase the capability to detect low PrPsc concentrations.

#### Electron microscopy on murine samples

Similar protocols for electron microscopy, conventional and with colloidal gold, as those described for bovine samples, were applied on murine samples. In summary, after homogenization in 10% sarcosyl in water during 30 seconds and N-octanol addition, serial centrifugations for 30 min at 22.000 g and 215.000 g in 1% sarcoyl and 10% NaCl were followed. Proteinase K incubation for 2 h at 37°C in the same buffer was applied on the provided pellet. Then, after centrifugation and removal of supernatant, the pellet containing the purified SAF protein was resuspended in distilled H_2_O. Touch grid technique previously described was applied in order to provide samples on formvar carbon grids. After soaking 1% SDS 1 min and rinsing in distilled water (x5), the grids were stained with 2% potassium phosphotungstate (pH 6.6) prior to TEM examination (80 KV).

#### Confocal microscopy on murine samples

With the purpose of identifying a specific cellular population that seemed to co-localize with PrPsc in several mice, and suggested to be a glial cell due to its morphology and distribution, a confocal microscopy study was carried out following the protocol previously established [28].

On this occasion, serial 50-μm sections were immersed in endogenous peroxidase (1%, 30 min) and Triton x-100 (0.1%, 3 hours). Pre-treatment, consisting of incubation with 98% formic acid (15 min), proteinase K digestion (15 min, 37°C) and hydrated autoclaving (15 min for 80°C overnight, o.n.) was included. The sections were then incubated (4°C o.n. with agitation) with the corresponding monoclonal prion-specific antibody (6H4, 1/500, Prionics, Zurich, Switzerland or F89, 0.5 μg/ml, VMRD Inc, USA) and polyclonal GFAP (1/500, DAKO, Hamburg, Germany). Subsequently, Streptavidin-conjugated Alexa 594 (1/200, Invitrogen, Eugene, Oregon) and Alexa 488 (1/200, Invitrogen, Eugene, Oregon) were respectively used as secondary antibodies for 1 hour in darkness. Incubation for 1 hour with IgG anti-mouse biotin (1/100 Invitrogen, Eugene, Oregon) was performed prior to fluorochrome addition in order to enhance the fluorescent signal for prion protein.

Each section was assessed by confocal imaging. Serial confocal Z-stacks were taken on a Zeiss laser-scanning confocal microscope LSM 510 (Carl Zeiss MicroImaging, Germany) with 10x (NA 0.3) and 20x (NA 0.5) objectives.

The fluorescence emission resulted from excitation from 488-nm and 594-nm lasers and was detected with a two-channel multi-track configuration, using band-pass 505–530 nm and long-pass 615 nm filters, respectively. Z-stacks were finally combined in one stack for each sample and processed by Zen 2008 software (Carl Zeiss MicroImaging, Germany).

## Results

All bovine samples had presented non-conclusive results with the initially applied rapid technique, Prionics®-Check WESTERN test, since the resultant signal was evident but did not correspond with the typical glycoform pattern of a classical BSE agent. Additionally, HERDCHECK BSE-SCRAPIE Antigen Test Kit EIA from IDEXX Laboratories which were applied to all bovine samples before inoculation in mice provided negative results since they did not reach the cutoff of the technique (Table 1).

**Table 1:** Results provided for each of the five samples in each of the tests performed

**Sample**				**After purification sample and by applying modified techniques**		
	**Prionics®-check WESTERN test**	**EIA IDDEX (Cut off = 0.22)**	**Immunohisto chemistry**	**Immunocyto chemistry**	**Confirmatory Western blot**	**Electron microscopy**
439	Non-conclusive	0.045	Non- conclusive	Positive	Non- conclusive	Positive
440	Non-conclusive	0.049	Non- conclusive	Positive	Non- conclusive	Positive
441	Non-conclusive	0.052	Non- conclusive	Positive	Non- conclusive	Positive
442	Non-conclusive	0.056	Non- conclusive	Positive	Non-conclusive	Positive
447	Non-conclusive	0.053	Non- conclusive	Positive	Non- conclusive	Positive

**Figure 1:**
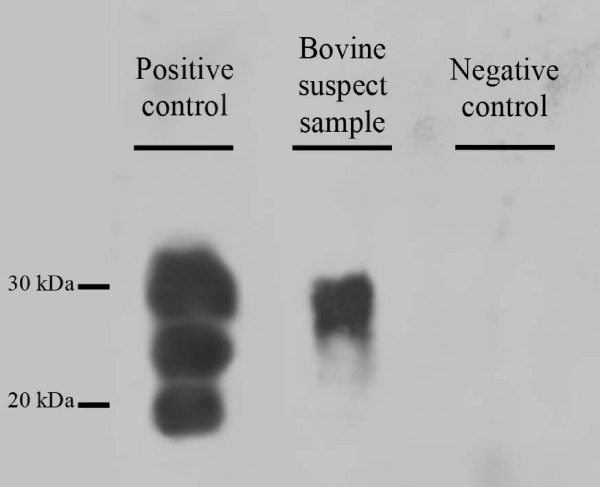
Western blotting corresponding to one of the bovine sample included in the study.

Because of the advanced autolysis, the histopathological examination was impossible. And for the other confirmatory techniques, not all of them offered a conclusive result. First, the result provided by Western blotting was non-conclusive because only a slight signal could be observed, even despite the additional protocol of PrPsc concentration and purification applied on the samples (Figure 1). In the same way, the Western blotting protocol for agent differentiation showed a similar non-conclusive result with both antibodies (results not shown).

**Figure 2:**
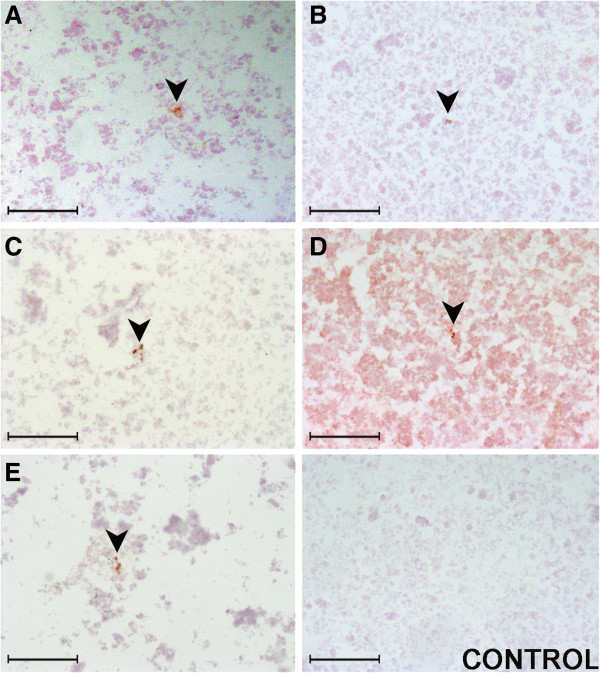
**Immunocytochemistry on bovine samples by using L42 antibody. (A**-**E)** From each one of the five samples included in the study; and from a healthy animal. Bar = 100 μm.

However, the immunocytochemistry applied on the bovine samples confirmed the positive diagnosis in all of them, because immunostaining could be observed in all the analyzed samples (Figure 2A - E). The immunostaining corresponded with PrPsc deposits because it did not appear in the negative controls and their presentation was repeated with the three different antibodies used for every sample. Finally, the modification in the protocol based on the inclusion of linker, did not improve the sensitivity of the technique for PrPsc detection.

**Figure 3:**
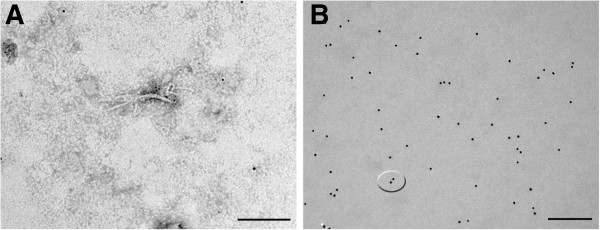
**SAF visualization by applying electron microscopy marked with colloidal gold (A; Bar = 0,2 μm) and paired particles of colloidal gold (B; Bar = 0,2 μm) in the nervous tissue of a bovine sample.** Colloidal gold particles = 10 nm.

These results corroborate those provided by electron microscopy which confirmed the positivity of all the samples since fibrils presenting SAF morphology and features were visualized in all of them. Moreover, the recognition of these fibrils by gold labelled specific primary antibodies confirmed their correspondence with PrPsc (Figure 3A). The appearance of these fibrils was similar to that showed by Narang and Perry [23] and Merz et al. [24], but they were present in a lower proportion and with a shorter length. Additionally, the colloidal gold was not only observed associated to these fibrils, but it also frequently appeared in pairs (Figure 3B). Specificity of colloidal gold particles was confirmed by total absence in negative autolytic samples.

Regarding results with the murine bioassay, several problems associated with the autolytic state of the samples appeared before the inoculation. All tested samples were heat treated to inactivate the microbial contamination and were inoculated after dilution (1.25 - 10%) to decrease the toxicity which could not be removed exclusively by the use of heat treatment. A high number of animals died during the 10 days post-inoculation period. A total of 229 mice were inoculated with the bovine homogenates, but only 148 animals survived and were finally included in the study. During the incubation period, 36 animals corresponding to the five bovine suspect samples showed clinical signs which corresponded with BSE symptoms like ataxia, loss of balance, loss of motor coordination and lordosis. Their appearance was late (18–24 months), even in the one animal which finally resulted positive by all tests applied (symptoms did not appear until 17 months). The earliest incubation period presented by a mouse with positive immunohistochemistry was 11 months; in the remaining samples, it was 15, 15, 17 and 19 months, respectively. Mean incubation (± SD) periods were 21.8 ± 2.9, 19 ± 1.8, 19 ± 4.5, 21.5 ± 2.2 and 18.6 ± 5.5, respectively.

Despite the remaining animals showed a negative result by the applied rapid test (Roboscreen®), some of them (73) showed a value not reaching cut off but slightly higher (0.02 - 0.058) than the presented by other animals (< 0.02). Specifically, all these 73 animals belonged to those 36 presenting clinical signs or had died before the end of the experiment due to reasons other than TSE (Table 2).

**Table 2:** Number of mice that presented clinical signs, resulted positive by immunohistochemistry and by Roboscreen® rapid test depending on the group of animals inoculated with homogenates from each bovine sample analyzed

**Bovine sample identification**	**Number of mice presenting clinical signs/ total number of inoculated mice**	**Number of mice with value higher than 0.02 by Roboscreen® test/ total number of inoculated mice**	**Number of mice IHC positive/ total number of inoculated mice**
**439**	**8 / 36**	**5 / 36**	**11 / 36**
**440**	**6 / 33**	**21 / 33**	**3 / 33**
**441**	**5 / 26**	**11 / 26**	**5 / 26**
**442**	**11 / 25**	**20 / 25**	**6 / 25**
**447**	**6 / 28**	**16 / 28**	**5 / 28**

**Figure 4:**
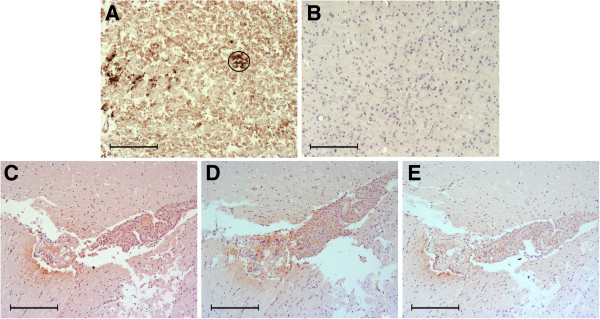
**PrPsc immunostaining in murine samples. (A)** Plaque found in the positive sample by Roboscreen test. **(B)** No immunostaining in a non inoculated animal **(C**-**E)** Granular staining by immunohistochemistry in one mouse corresponding to a remaining positive bioassay sample using the three different antibodies: 6H4 , R145, F89, respectively. Bar = 200 μm.

**Figure 5:**
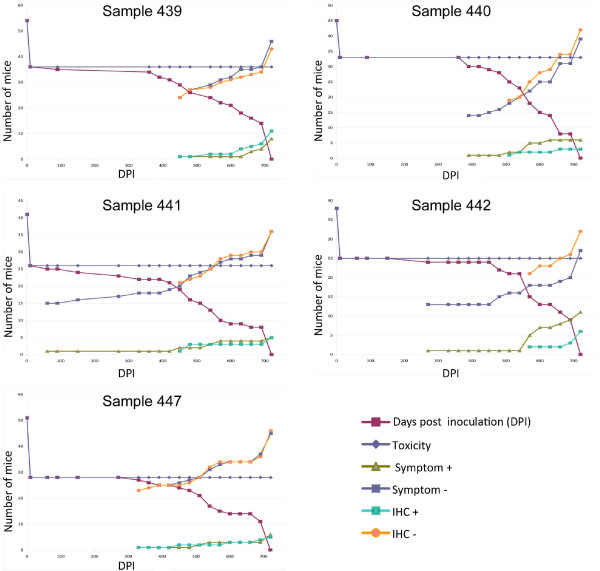
Graph illustrating the survivals of the individual mice in each of the five groups showing those mice which died from inoculum toxicity or presenting specific clinical signs of prion disease and those which were positive by immunohistochemistry.

The immunohistochemical protocol applied to murine samples evidenced a PrPsc plaque pattern in only the one animal which had a positive result using the Roboscreen® test (Figure 4A). However, 18 inoculated animals (distributed in all the groups corresponding to every bovine sample analyzed) presented granular deposits in some of the 5 sections examined, by applying the three antibodies used (Figure 4B - D). Moreover, 12 mice showed similar deposits by applying two antibodies (being impossible to obtain serial sections in these cases for the third antibody tested). As a consequence, a total of 30 animals were considered as positive by immunohistochemistry. It is relevant to point out that variations applied to this protocol consisting of the addition of the linker or the replacement by an ultrasensitive immunohistochemistry did not increase the sensitivity of the technique since they did not detect a higher number of positive animals. The correlation between animals which presented clinical signs and positive mice by immunohistochemistry was found (Figure 5) in all cases except for those mice that had prematurely died owing to other causes than BSE (showing PrPsc deposits in different areas of the brain but not TSE symptoms).

**Figure 6:**
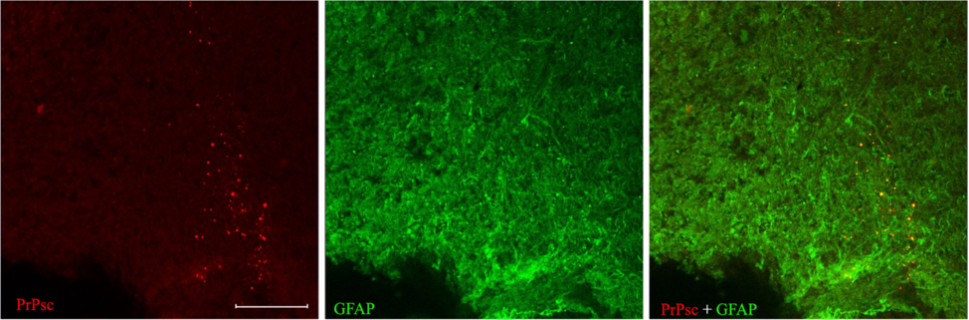
**Co-localization of PrPsc deposits (red) and glial cells (green) shown by confocal microscopy (yellow).** Bar = 200 μm.

An interesting finding worthy of mention is that PrPsc deposits seemed to be associated with a specific glial cell population in some areas from some sections (mouse brain). The confocal microscopy technique, applied in order to identify this cellular type, demonstrated an evident co-localization between PrPsc accumulation and astrocytes (Figure 6).

**Figure 7:**
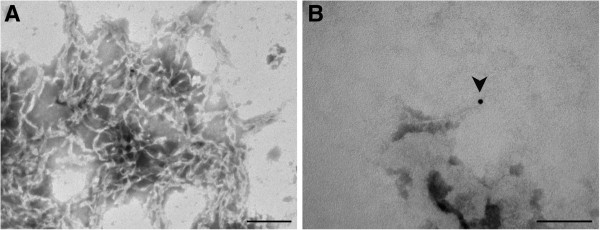
**Fibrils visualized by electron microscopy. (A)** In the positive mouse by Roboscreen® test (Bar = 0,2 μm) **(B)**. In a negative by Roboscreen® test but positive by immunohistochemistry, marked with colloidal gold (Bar = 0,1 μm).

As it happened in bovine samples, results provided by immunohistochemistry on murine samples were confirmed by visualization of fibrils by electron microscopy. Fibrils not only appeared in the mouse brain found positive by the Roboscreen® test but also in other mice which had positive immunohistochemical results but were negative with the rapid tests. A clear difference relative to the proportion of fibrils visualized, much higher in the positive animal by rapid test than in the rest of them where only isolated fibrils were found, was evidenced (Figure 7A). Even so, regardless the proportion the appearance and the size coincided with those fibrils which had been observed in the bovine samples. Particles of colloidal gold recognizing these fibrils could unequivocally confirm the positive diagnosis of all the samples (Figure 7B).

Additionally, a second passage using brain material from the only mouse showing a Roboscreen® positive and immunodeposits in plaque provided positive results by this same technique as well as clinical signs within 7–8 months post-inoculation in all mice inoculated (data not shown). These results would support the theory based on a low PrPsc concentration in the sample which might explain the variability of the results.

## Discussion

All the studied bovine samples were confirmed as BSE positive cases thanks to the results provided by immunocytochemistry, electron microscopy and bioassay. Despite all the applied techniques being standardized for increasing their capability to detect PrPsc, the extremely scarce concentration of PrPsc in the samples is proposed here as the most coherent reason why non-conclusive results (weak signal presenting an unconventional pattern) were provided by Western blotting as well as showing different titers in the bioassay (reflected by the lack of 100% success of transmission in all the mice included in it). Therefore, the positive results provided by immunohistochemistry on murine bioassay ultimately demonstrated that initial samples were infectious and transmitted the disease. This was also concluded by immunocytochemistry and SAF detection by electron microscopy being applied on the initial samples. However, rapid tests and immunoblotting did not result useful for convincingly confirming these results.

Although rapid techniques applied in this study achieved their objective as screening tests by showing a non-conclusive signal in the samples analyzed, they were not able to give definite results, demonstrating a lower sensitivity compared to some of the confirmatory techniques (not all in this occasion, since immunoblotting, despite the combined protocol described, was not able to improve the results provided by rapid tests in terms of sensitivity). Non-conclusive diagnosis obtained by these techniques in the bovine as well as false negative diagnosis in the murine samples have been previously justified on the basis of the low PrPsc concentration and the unsuitable selection of area to be analyzed [29]. Both are unavoidable and intrinsic features for the samples studied here. Overall, these findings might call into question the recent proposal determined by the OIE which dictates that two equal results with two different rapid techniques are enough to give an official BSE confirmation [30]. Furthermore, the results of this study confirm immunocytochemistry as a useful and rapid tool for diagnosis in liquid state samples, solving a relevant problem in BSE and Scrapie epidemiology.

As it had been previously impossible to apply histopathological examination due to the advanced degree of autolyisis of the analyzed samples, immunohistochemistry was evidenced as the first and reliable technique to diagnosis of samples included here. Immunocytochemistry has been demonstrated here as a technique capable to provide final results for bovine samples. Moreover, this study first demonstrates the reliability of this technique established by Monleón et al. [18] since their results have been corroborated by other confirmatory techniques, specifically, by electron microscopy and by *in vivo* tools (bioassay). Confirmation of the immunocytochemical positive results by electron microscopy clearly discards the possible subjectivity and non-specificity which some authors could state for the immunocytochemical assessment established, since SAF visualization has been demonstrated to be exclusive to TSEs [31]. In spite of the proportion and length of fibrils being lower than those previously described in conventional positive cases [32], the immunolabelling of these fibrils by colloidal gold [33] unequivocally identified PrPsc in all of them. This slight difference in appearance is probably associated with the low PrPsc concentration assumed at the beginning and finally evidenced in this study. Besides, the visualization of paired gold deposits which seem to correspond with PrPsc dimers described by Dourmashkin et al. [34], supports this hypothesis, associating gold deposits to areas of potential formation of fibrils [35]. As a consequence, although it is a technique currently fallen into disuse, SAF visualization by electron microscopy is confirmed in this study as a technique with high sensitivity despite PrPsc concentration and autolysis of the sample, demonstrating to be useful for diagnosing this kind of samples.

On the other hand, although the bioassay presented a transmission rate lower than expected (only 24% of the inoculated mice developing clinical signs), its sensitivity was 100% since it confirmed the positive diagnosis of the bovine samples. The low number of affected mice, expressed in the low percentage of animals which presented clinical signs or positive result by diagnostic techniques, could be explained by several reasons. Despite partly avoiding the problem of species barrier by use of bovinized transgenic mice [36], the autolytic state of samples could have hampered the choice of the area of study where PrPsc was present. Nevertheless, the main responsible factor for this failure seems to be the low initial PrPsc concentration due to, advancement of the protease digestion associated with the putrefaction process, the serial dilutions and heat treatment which had to be applied on the samples before inoculation. This scarce PrPsc presence could elongate the incubation period and, indirectly, cause false negatives in those mice which died prematurely, by natural death or euthanasia, before presenting clinical signs [37] or not presenting enough concentration of PrPsc to be detected by the applied techniques at that moment [38]. The fact that the only mouse clearly considered positive by rapid tests and the only mouse showing PrPsc plaques (as usually seen in BSE inoculated mice) [39] belonged to the group of animals where the highest concentration was used for inoculation, confirms this theory.

Moreover, the presence of isolated fibrils in the mice analyzed [40] instead of grouped fibrils visualized by electron microscopy in the only positive mouse by rapid tests [41], would, once again, correlate with this hypothesis.

As for the variance between the presence of PrPsc deposits and presentation of clinical signs found in some mice, PrPsc concentration used for inoculation could affect the results but also other explanations arise on this matter [42]. The possibility of a transmission of the disease without neurological signs [43] or a possible non-exclusive relationship between PrPsc and infectivity [44-46] and neurodegeneration [47], among them. Besides, the demonstration of the existence of animals as persistent carriers [48] or with a subclinical state of the disease [47], already described in mice with high titers of infection but no symptoms [49], should be borne in mind. This same lack of correlation between PrPsc and infectivity could be reflecting in the animals with symptoms but no PrPsc deposits [50], considering in this case that the disease might be transmitted without detectable PrPsc [44], even with high titers [51].

Further studies with non-diagnostic aims but for identification of astrocytes co-locating with PrPsc were developed here, as mentioned in Material and Methods section. The relationship between PrPsc and glial cells observed by conventional immunohistochemistry, was confirmed by confocal microscopy showing an evident co-localization. This finding would put in evidence the possibility of the participation of these cells, possibly across the haematoencephalic barrier [27,52], in the prion propagation in the model used here [53].

On this occasion, it was not possible to associate the studied samples to classical or atypical BSE owing to the lack of success to provide a clear banding pattern by Western blotting and/or a characteristic profile by immunohistochemistry, the lack of reference to previous data on incubation periods and the inability to determine lesion profiles in the mouse line. All these facts, as it has been stated, probably due to the very scarce concentration of PrPsc. Otherwise, whether the agent present in samples was atypical BSE or classical BSE with really low PrPsc deposits and low infectivity titers was not possible to be determined.

## Conclusions

In conclusion, demonstration of transmission of the disease even with low concentrations of PrPsc [54], highlights BSE’s ability to adopt different behavior, even sometimes similar to Scrapie [55], should reinforce that vigilance is required in interpreting results so that subtle changes do not go unnoticed. Additionally, to maintain a continued supervision of the techniques which are applied in the routine diagnosis would prove essential for the ultimate eradication of the disease. A study of the actual BSE presence should be considered as necessary because a state of sporadic prevalence could exist [56] and samples without a diagnosis [57,58] could reach the food chain, involving therefore a risk for public health.

## Competing interests

The authors declare that they have no competing interests.

## Authors’ contributions

RS carried out all experimental studies except for murine assay. DB was responsible for developing of the overall bioassay. JB helped to draw conclusions and write the manuscript. MM designed the study, supervised the experimental studies and results provided and wrote the manuscript. All authors read and approved the final manuscript.
